# Hyperemesis in Pregnancy: Complications and Treatment

**DOI:** 10.3390/medsci13030132

**Published:** 2025-08-14

**Authors:** Angeliki Gerede, Sofoklis Stavros, Efthalia Moustakli, Anastasios Potiris, Ilias Orgianelis, Athanasios Zikopoulos, Peter Drakakis, Ekaterini Domali, Makarios Eleutheriades, Nikolaos Nikolettos

**Affiliations:** 1 Department of Obstetrics and Gynecology, Democritus University of Thrace, 69100 Alexandroupolis Campus, Greece; eliasnelis@gmail.com (I.O.); nnikolet@med.duth.gr (N.N.); 2 Third Department of Obstetrics and Gynecology, University General Hospital “ATTIKON”, Medical School, National and Kapodistrian University of Athens, 12462 Athens, Greece; sfstavrou@med.uoa.gr (S.S.); apotiris@med.uoa.gr (A.P.); thanzik92@gmail.com (A.Z.); pdrakakis@med.uoa.gr (P.D.); 3 Laboratory of Medical Genetics, Faculty of Medicine, School of Health Sciences, University of Ioannina, 45110 Ioannina, Greece; ef.moustakli@uoi.gr; 4 First Department of Obstetrics and Gynecology, Alexandra Hospital, Medical School, National and Kapodistrian University of Athens, 11528 Athens, Greece; kdomali@yahoo.fr; 5 Second Department of Obstetrics and Gynecology, University Hospital “Aretaieion”, Medical School, National and Kapodistrian University of Athens, 11528 Athens, Greece; melefth@med.uoa.gr

**Keywords:** hyperemesis, nausea, vomiting, pregnancy, nutrition, prognosis

## Abstract

Background: Hyperemesis gravidarum (HG) is the leading cause of hospitalization during early pregnancy, affecting approximately 0.3–3% of pregnancies. It represents the most severe end of the nausea and vomiting in pregnancy (NVP) spectrum and is associated with substantial maternal morbidity and potential adverse fetal outcomes. Despite extensive research, the exact pathophysiology remains poorly understood, and optimal management strategies continue to be debated. Methods: This narrative review synthesizes current evidence on the complications and treatment approaches for HG. A literature search was conducted in PubMed, Scopus, and Medline up to October 2024 using predefined keywords. Eligible sources included observational studies, cohort studies, descriptive studies, and case reports. Systematic reviews, meta-analyses, and non-English articles were excluded. Results: HG is associated with a broad spectrum of complications, including dehydration, electrolyte imbalances, Wernicke’s encephalopathy, cardiac arrhythmias, thromboembolism, and adverse pregnancy outcomes such as fetal growth restriction and preterm birth. Pharmacological treatments—most notably doxylamine-pyridoxine (the only FDA-approved therapy), ondansetron, metoclopramide, and corticosteroids—have demonstrated varying efficacy and safety profiles. Non-pharmacological interventions such as acupressure, dietary adjustments, psychotherapy, and hypnosis have also been studied, although evidence remains limited. Conclusions: HG requires a comprehensive and individualized management approach. While doxylamine-pyridoxine remains the cornerstone of therapy, other pharmacologic and supportive measures may offer additional benefit. Continued research is essential to clarify the underlying mechanisms, improve therapeutic efficacy, and develop evidence-based guidelines that integrate both medical and psychosocial care for affected women.

## 1. Introduction

Hyperemesis gravidarum (HG) is a severe and persistent form of nausea and vomiting during pregnancy, affecting 0.3–2% of pregnant women [1]. While nausea and vomiting during pregnancy (NVP) are common, experienced by up to 75% of pregnant women, HG represents the extreme end of this spectrum. Common symptoms include ptyalism (excessive salivation), fatigue, weakness, and dizziness, while less common manifestations may involve hyperolfaction, dysgeusia (altered taste perception), sleep disturbances, depression, anxiety, irritability, and mood swings [2]. Severe cases often lead to hospitalization due to complications such as weight loss, ketonuria, dehydration, electrolyte imbalances, acid-base disturbances, and arrhythmias [1,3].

Several risk factors have been associated with HG, including first-trimester pregnancy, primigravidity (first pregnancy), multiple gestation, obesity, a family history of HG, trophoblastic disease, hyperthyroid disorders, psychiatric conditions, and molar pregnancy [2,3,4]. The complications of HG can be categorized into three main groups. Pregnancy-related complications include malnutrition, anemia, hyponatremia, Wernicke’s encephalopathy, kidney failure, central pontine myelinolysis, stroke, cerebral artery vasospasms, seizures, coagulopathy, hypoglycemia, esophageal rupture or perforation, liver disease, jaundice, pancreatitis, deep vein thrombosis, pulmonary embolism, pneumothorax, pneumomediastinum, rhabdomyolysis, vitamin K deficiency, splenic avulsion, depression, and post-traumatic stress disorder. Central nutrition-related complications encompass sepsis, fungemia, tamponade, local infection, venous thrombosis, fatty infiltration of the placenta, and transaminitis. Infant-related complications include low birth weight, small-for-gestational-age status, and preterm birth before 37 weeks [5,6,7,8]. Some authors have investigated whether other gynecological conditions might influence HG risk, but to date there is no clear evidence. For instance, common disorders such as polycystic ovary syndrome or endometriosis have not been shown to increase HG risk beyond known factors (e.g., trophoblastic disease) [9]. HG remains primarily associated with placental and hormonal conditions [10].

The prevalence of HG varies globally, with reported rates of 3.6% in Malaysia and Eastern Asia, 4.4% in Ethiopia, 10.8% in China, and 0.3% in Sweden [11]. Early identification and aggressive treatment are essential to mitigate maternal and fetal risks. However, both pregnant women and healthcare providers often exhibit hesitancy in using pharmacological treatments due to concerns about fetal safety. The historical thalidomide tragedy, in which babies were born with severe limb deformities after in utero exposure, has reinforced fears regarding the teratogenic risks of pregnancy medications. While several available treatments offer symptom relief, many patients fail to achieve adequate control, emphasizing the need for novel and more effective therapeutic strategies [12]. The purpose of this narrative review is to provide a comprehensive overview of the complications associated with HG and to evaluate current and emerging treatment strategies. We aim to inform clinicians of evidence-based management options and to identify gaps for future research.

## 2. Materials and Methods

This narrative review explores the challenges, complications, and treatment approaches for HG during pregnancy. Using the databases Scopus, Medline, and PubMed, a thorough literature analysis was carried out to find pertinent research published up until October 2024. Relevant articles were retrieved using keywords such as “Hyperemesis gravidarum,” “treatment,” “complications,” and “management.” This review includes case reports, descriptive studies, observational studies, and cohort studies that addressed the pathogenesis, treatment approaches, and related consequences of HG. To ensure a well-rounded conversation, other sources were explored, such as expert viewpoints and therapeutic guidelines. Articles not published in English and those focusing on non-pregnant populations were excluded. The exclusion of non-English language studies was due to logistical constraints, including the unavailability of professional translation services and the need to ensure consistency and accuracy in data extraction. However, we recognize this introduces the possibility of language bias and may have resulted in the omission of relevant international literature.

**Figure 1:**
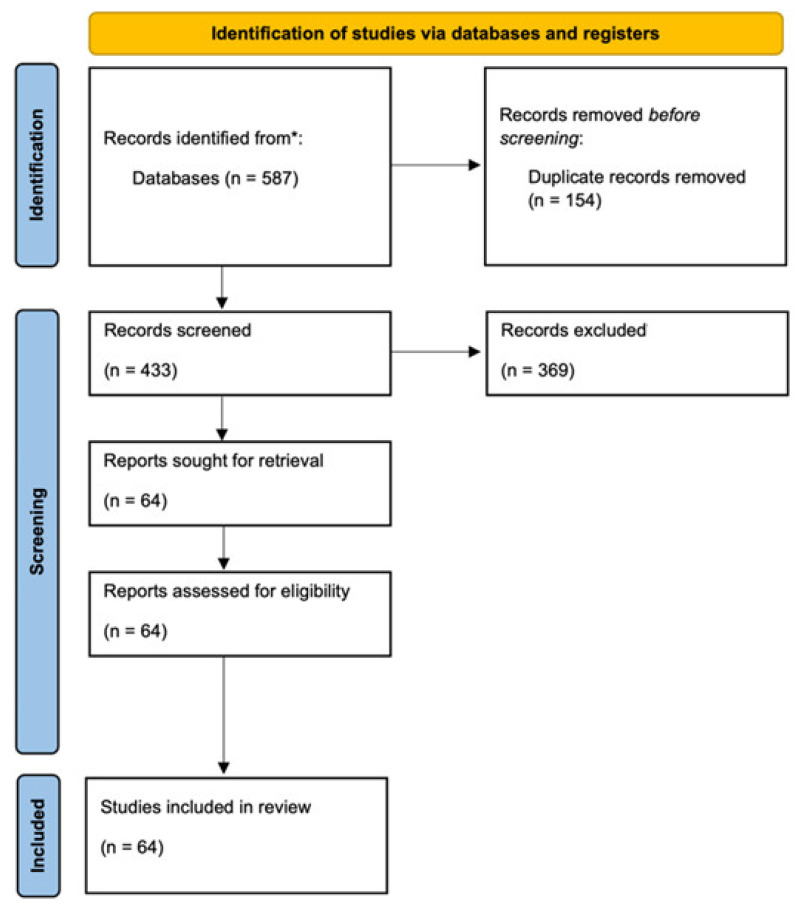
Flow diagram of the included studies. * Scopus, Medline, and PubMed.

To ensure relevance to the research issues, the chosen studies were assessed using the PICOS criteria (Population, Intervention, Comparison, Outcome, and Study design). A comprehensive overview of existing knowledge, ongoing issues, and potential research paths in HG management was provided by synthesizing findings from a variety of study types, despite the fact that a structured risk-of-bias evaluation was not conducted because of the narrative style of this review. Nevertheless, we qualitatively assessed key studies. Major trials are identified as randomized or observational, and we note any obvious limitations (e.g., small sample size) when summarizing their findings to provide context on evidence quality. Figure 1 shows a flowchart of the literature search, screening, and selection process (PRISMA diagram) used for this review.

## 3. Results

### 3.1. Overview of Existing Literature

A comprehensive review of the literature identified 64 relevant studies discussing HG, its complications, and treatment approaches. Risk factors, pathophysiology, pharmaceutical and non-pharmacological therapies, and maternal-fetal outcomes were among the many facets of HG that were examined in these studies. The effectiveness of corticosteroids, alternative therapies, antiemetic medications, and nutritional therapy has been studied to differing degrees. Furthermore, a number of research studies have emphasized the neurological, cardiovascular, and psychological issues linked to severe HG patients.

The variety of HG research was reflected in the selected studies, which included observational research, case reports, cohort studies, and randomized controlled trials (RCTs). Concerns regarding the safety and effectiveness of pharmacological therapies, such as corticosteroids, ondansetron, and doxylamine-pyridoxine, persist despite extensive research. Although they have been studied, non-pharmacological strategies like acupressure, psychotherapy, and dietary changes lack strong clinical support. An overview of the main conclusions from a few chosen studies is presented in Table 1.

**Table 1:** Overview of studies on treatments for HG.

Study	Type of Study	Type of Treatment/Intervention
[13]	RCT	Doxylamine-pyridixonine
[14]	RCT	Doxylamine-pyridixonine
[15]	RCT	Doxylamine-pyridixonine
[16]	CSS	Cyclizine
[17]	RCT	Promethazine
[18]	CS	Cyclizine, Promethazine
[19]	RCT	Metoclopramide
[20]	CS (retrospective)	Metoclopramide
[21]	CS	Ondansetron
[22]	DS	Prednisolone
[23]	DS	Methylpresnisolone
[24]	CaS	Cannabis
[25]	CS (retrospective)	Cannabis
[26]	OS	Ginger and Vitamin B6
[27]	RCT	Acupressure
[28]	RCT	Psychotherapy
[29]	CaS	Hypnosis
[30]	RCT	Hypnosis
[31]	RCT	Mirtazapine
[32]	RCT	Capsaicin
[33]	RCT	Metoclopramide, ondansetron
[34]	RCT	Diazepam
[35]	RCT	Granisetron, promethazine
[36]	RCT (retrospective)	Hydrocortisone, metoclopramide
[37]	RCT	Acupressure
[38]	RCT	Outpatient care, Inpatient care
[39]	RCT	Holistic individual care/Standard care
[40]	CS	Proton pump inhibitors
[41]	RCT	Oral rehydration
[42]	RCT	Watermelon in diet after hospital discharge
[43]	RCT	Gabapentin
[44]	RCT	Ondansetron, metoclopramide
[45]	RCT	Transdermal clonidine

CS: Cohort study, CaS: Case study, OS: Observational study, DS: Descriptive study, RCT: Randomized controlled trial.

### 3.2. Etiology and Pathophysiology

HG commonly begins between weeks 4 and 5 of pregnancy and is most severe during the first trimester [46]. Although its exact pathophysiology remains unclear, multiple mechanisms have been proposed, including hormonal, immune, gastrointestinal, and psychological factors [47]. Elevated levels of growth differentiation factor 15 (GDF15) have been implicated in HG, as increased GDF15 levels under dietary stress may send an endocrine signal to the brain, triggering nausea, weight loss, and muscle wasting, similar to cachexia seen in tumor-bearing patients [48,49]. Studies have shown significantly higher circulating GDF15 levels in women hospitalized with HG, reinforcing this potential link [50]. Additionally, human chorionic gonadotropin (hCG) has been associated with the onset of HG symptoms, with research indicating significantly elevated serum hCG levels in pregnant women diagnosed with HG [51]. Other findings suggest that hCG stimulates thyroid hormone production, contributing to temporary hyperthyroidism, which has been frequently observed in HG patients [52].

Gastrointestinal motility dysfunction has also been suggested as a contributing factor. Increased levels of progesterone and estrogen during pregnancy may disrupt gastric slow-wave rhythms, leading to delayed gastric emptying and worsening nausea and vomiting. Some studies have also proposed that an overactive maternal immune response may play a role, as pregnant women with HG have shown higher levels of fetal cell-free DNA, suggesting trophoblast damage and maternal immune overactivation. Inflammation may further contribute to HG, with tumor necrosis factor-alpha (TNF-α) being identified as a potential mediator. Chronic *Helicobacter pylori* (*H. pylori*) infection has also been investigated as a risk factor, as many HG patients test seropositive for the bacterium. However, the correlation between *H. pylori* infection and HG symptom severity remains unclear.

Psychological factors are another area of interest, as studies have found that women with HG are more likely to have a history of anxiety or mood disorders before pregnancy. Anxiety or depression requirements were met by 57.4% of women with HG, according to prospective research; some of them met both criteria. A different study also found that one-third of HG patients had previously had a psychiatric diagnosis, indicating that psychological stress may make symptoms worse. Although more investigation is required to determine clear processes, the interaction of serotonin, dopamine, and gut–brain signaling in the regulation of nausea is also being investigated.

### 3.3. Complications Associated with HG

Significant HG cases have been linked to a number of side effects, such as systemic, cardiovascular, and neurological consequences. Prolonged electrolyte imbalances and dehydration may raise the risk of neurological complications such as Wernicke’s encephalopathy (WE), central pontine myelinolysis (CPM), and venous thrombosis, according to research [2]. Several studies suggest that intravascular dehydration brought on by HG may increase the risk of stroke by causing venous thrombosis, especially in people who have underlying vascular abnormalities, such as developmental venous abnormality (DVA). Due to its non-specific symptoms, WE is still underdiagnosed, with over 80% of cases being either misdiagnosed or not recognized, despite being one of the most commonly reported neurological consequences of HG [53,54].

The majority of the data regarding HG’s cardiovascular effects comes from case reports rather than extensive research [55,56,57]. In HG patients, case reports have reported ventricular arrhythmias, QT prolongation, and significant blood pressure swings, which are frequently linked to electrolyte abnormalities such as hypokalemia. Persistent metabolic abnormalities during pregnancy may lead to future cardiovascular risks, according to a countrywide cohort study that found a possible association between HG and long-term maternal cardiovascular morbidity [58]. The complications outlined in Table 2 highlight the diverse effects of HG on maternal health (Table 2). To prove a conclusive relationship, more research is necessary because there are not many large prospective studies available.

In addition to maternal sequelae, severe HG has been increasingly associated with adverse pregnancy outcomes. Multiple cohort studies have demonstrated that women hospitalized for HG are at elevated risk for preterm birth, intrauterine growth restriction (IUGR), and low birth weight infants. A large population-based cohort study in Sweden by Fiaschi et al. (2018) found that HG was significantly associated with small-for-gestational-age (SGA) births, low birth weight (<2500 g), and preterm delivery (<37 weeks), even after adjusting for confounding factors [59]. These findings were corroborated in subsequent studies from Norway and Canada, which also linked HG with increased risk of placental dysfunction disorders, including preeclampsia and placental abruption [60,61].

**Table 2:** Overview of complications associated with HG.

Study	Type of Study	Complications
[61]	CSr	Adverse pregnancy outcomes
[62]	CaS	Intracerebral hemorrhage
[63]	CaS	Sagittal sinus thrombosis
[64]	CaS	Simultaneous optic neuropathy and osmotic demyelinating syndrome
[65]	CaS	Wernicke encephalopathy and central pontine myelinolysis
[66]	CaS	Wernicke encephalopathy (WE)
[57]	CaS	Severe epigastric pain
[56]	CaS	Shortness of breath, palpitations, and atypical chest tightness
[55]	CaS	Ventricular tachycardia
[60]	CaS	Placental dysfunction disorders
[58]	CaS	Long-term cardiovascular morbidity
[67]	CaS	Postural hypotension and autonomic neuropathy
[68]	CaS	Pneumomediastinum following esophageal rupture
[69]	CaS	Diaphragmatic tear
[59]	CaS	Perinatal and postnatal complications
[70]	CaS	Rhabdomyolysis
[71]	CaS	Rhabdomyolysis and diabetes insipidus
[72]	CaS	Cardiac arrest
[73]	CaS	Gitelman syndrome-associated hypokalemia and hypomagnesemia
[74]	CaS	Vitamin K deficiency embryopathy
[75]	CaS	Vitamin K deficiency
[76]	CaS	Vitamin K deficiency-induced fetal intracranial hemorrhage and hydrocephalus
[77]	CaS	Brachytelephalangic chondro-dysplasia punctata and gray matter heterotopias
[78]	CaS	Vitamin K deficiency embryopathy
[79]	CaS	Fetal intracranial hemorrhage associated with vitamin K deficiency
[80]	CaS	Hyper-parathyroid crisis
[81]	CaS	Transient thyrotoxicosis
[82]	CaS	Candida septicemia
[83]	CaS	Pulmonary embolism
[84]	CSr	Depression, anxiety, post-traumatic stress

CS: Cohort Study, CSr: Cohort Study Retrospective CaS: Case Study, OS: Observational Study, DS: Descriptive Study, SCS: Single-Center Study, RT: Randomized Trial.

### 3.4. Treatment of HG

Several treatments have been studied for HG and nausea and vomiting during pregnancy. A delayed-release combination of pyridoxine hydrochloride (10 mg) and doxylamine succinate (10 mg) is the only treatment specifically indicated for pregnancy-associated nausea and vomiting in Canada and the United Kingdom. Randomized controlled trials have demonstrated that this combination is more effective than placebo in reducing nausea and vomiting in pregnant women [13]. However, the clinical significance of these results remains uncertain due to the small sample sizes of some studies.

First-generation antihistamines, such as cyclizine, dimenhydrinate, and promethazine, have been evaluated in several studies for their efficacy in managing pregnancy-related nausea and vomiting. While these medications have not shown superior effectiveness compared to placebo or other treatments [85], some studies suggest they may offer additional anticholinergic benefits for managing hypersalivation during pregnancy [86].

The safety of metoclopramide has largely been resolved, but concerns regarding the potential risks associated with ondansetron, specifically the possibility of heart abnormalities and cleft palate, remain inconclusive [87]. The routes of administration for treatments like metoclopramide and ondansetron (e.g., sublingual, rectal) have not been thoroughly studied in the context of HG, although similar treatments for other vomiting conditions have demonstrated comparable efficacy [87].

Intravenous fluid therapy has been found to be an effective treatment for dehydration due to nausea and vomiting, and recent studies show that outpatient intravenous administration of sodium chloride and potassium chloride is just as effective as inpatient treatment, while also reducing hospital stay duration [88].

Studies on dietary interventions suggest that low-energy, high-protein meals may help reduce nausea and vomiting in pregnant women, as opposed to high-carbohydrate diets [89]. Psychosocial support has also been found to alleviate the emotional and psychological impacts of HG, particularly for women facing difficulties with daily tasks such as child care [90].

Regarding other treatments, the impact of *H. pylori* eradication on HG symptoms remains unclear [91]. Proton pump inhibitors and antidepressants are suggested for managing specific symptoms such as constipation and psychological distress [92]. Additionally, while cannabis has been shown to have antiemetic effects in chemotherapy patients [93], its use during pregnancy has been linked to adverse outcomes for neurodevelopment in children [24,25]. Cannabis use is explicitly contraindicated in pregnancy due to evidence of fetal neurodevelopmental harm [22]. Similarly, unproven complementary therapies (e.g., herbal or homeopathic remedies) lack sufficient evidence and are not recommended for HG management. We emphasize that none of these alternative or complementary therapies should be used in place of evidence-based treatments. For example, cannabis is explicitly discouraged [22], and herbal/homeopathic remedies lack proven benefit for HG.

Current guidelines (e.g., ACOG Practice Bulletin; RCOG Green-top Guideline) recommend the delayed-release doxylamine–pyridoxine combination as first-line therapy for HG. Secondary options include antihistamines (such as diphenhydramine or dimenhydrinate) and dopamine antagonists (e.g., metoclopramide or ondansetron), with corticosteroids reserved for refractory cases. Table 3 summarizes the preferred treatments for HG, ranked by efficacy and safety according to these recommendations.

**Table 3:** Current therapeutic approaches for HG.

Therapeutic Approach	Treatment Class	Example Treatments	Clinical Efficacy	Safety Profile	Guideline/Evidence Support	Study
First-line	Antiemetics: H1-antagonists + B6	Doxylamine–pyridixonine	Moderate to high	Excellent (FDA Category A)	ACOG and RCOG recommend as first-line therapy	[7,94]
	Antihistamines (H1-blockers)	Diphenhydramine, Meclizine, Dimenhydrinate	Moderate	Generally safe (sedation common)	Widely used in early pregnancy per RCOG	[94]
	Nutritional/dietary interventions	Ginger supplements, frequent small meals	Low to moderate	Very safe	RCOG supports ginger for mild NVP	[94]
Second-line	Dopamine antagonists	Metoclopramide, Promethazine, Prochlorperazine	Moderate to high	Moderate (extrapyramidal risk)	Recommended if first-line fails; use cautiously	[94,95]
	5-HT_3_ receptor antagonists	Ondansetron	High	Generally safe; minor controversy	RCOG supports ondansetron after first-line failure	[94,96]
Third-line/Refractory	Corticosteroids	Methylprednisolone, Dexamethasone	Moderate	Risk of cleft palate in 1st trimester	Use cautiously in refractory HG after 10 weeks.	[94,97]
	Off-label adjuncts	Mirtazapine	Emerging; case-based	Appears safe in limited data	Limited data; small series support use in refractory HG	[98]
Adjunctive/Supportive	IV hydration and electrolyte therapy	IV fluids + potassium correction	Essential	Safe if monitored	Core to HG care; prevent renal/cardiac issues	[94]
	Thiamine supplementation	Oral or IV thiamine	Prevents Wernicke’s encephalopathy	Very safe	Strongly advised with prolonged vomiting	[94]
	H_2_ blockers/PPIs	Ranitidine, Omeprazole	Low (symptomatic relief only)	Safe in pregnancy	Manage concurrent gastritis or GERD	[94]
	Non-pharmacologic adjuncts	Acupressure, CBT, hypnosis	Low to moderate	Very safe	Complementary options; may relieve symptoms in some women	[94,99]
	Nutritional support (enteral/TPN)	NG/PEG feeding, parenteral nutrition	Necessary in severe HG	Moderate (risk of infection/metabolic effects)	For refractory malnutrition under close supervision	[94]

### 3.5. Management and Prevention

Research has shown that comorbidities can significantly impact the course of HG. For instance, conditions such as diabetes, epilepsy, Human Immunodeficiency Virus (HIV), autoimmune diseases, and mental disorders can complicate the management of HG, particularly when oral intake is insufficient [100,101]. Pregnant women with a history of bariatric surgery should be evaluated for potential intestinal herniation if they present with vomiting and abdominal pain, as they may be at increased risk for bowel obstruction due to nutritional deficiencies and impaired absorption of medications [100]. The recurrence of HG in subsequent pregnancies has been well-documented, with recurrence rates ranging from 15% to 86%. Studies that rely on hospital admissions report lower recurrence rates compared to those based on maternal self-reporting of symptoms and treatment needs [101].

One randomized trial suggests that early administration of pyridoxine hydrochloride and doxylamine succinate may reduce the severity and duration of pregnancy-related nausea and vomiting when used preventatively after pregnancy begins [102]. However, additional studies are required to confirm the effectiveness of this approach.

## 4. Discussion

The findings of this review align with the growing body of literature indicating that HG remains a significant clinical challenge with profound implications for both maternal and fetal health. Although the pathophysiology of HG is still complicated and poorly understood, this study emphasizes the significance of a number of putative causes, such as immunological responses, variations in hormones, gastrointestinal motility, and psychological variables. Elevated levels of GDF15 and hCG have been connected to the pathophysiology of HG. The results of current research suggest that hCG may be linked to temporary hyperthyroidism in impacted individuals and elevated GDF15 levels in HG-afflicted women. The hypothesis that HG may be caused by a mix of immunological and endocrine disorders is supported by these studies.

Intestinal disruption has also been connected to HG; studies suggest that elevated progesterone and estrogen levels may delay stomach emptying and enhance symptoms. This is an area where further research is needed to better understand the mechanisms of gastrointestinal motility in HG and how it could be addressed in treatment strategies. Further research is essential to determine the exact role that stress and gut–brain signaling play in the development and intensity of symptoms, even if psychological factors like anxiety and mood disorders seem to make HG worse. Thus, in accordance with the psychosocial interventions recommended in the literature, a multidisciplinary strategy that includes psychological assistance may be helpful for patients with severe HG.

The examination of HG-related consequences has shed light on a number of important topics, most notably the elevated risk of neurological disorders such as central pontine myelinolysis and Wernicke’s encephalopathy. These results are consistent with previous studies that show the impact of electrolyte imbalances and chronic dehydration in the development of these detrimental consequences. Wernicke’s encephalopathy in particular continues to be underdiagnosed, with many instances going unnoticed because of the symptoms’ lack of specificity. Clinicians need to be on the lookout for these consequences, particularly in severe HG cases when dehydration is widespread. Given that early evidence indicates possible long-term maternal cardiovascular hazards associated with chronic metabolic abnormalities throughout pregnancy, our findings further underscore the necessity for additional investigation into the cardiovascular implications of HG. The sole FDA-approved medication for pregnancy-related nausea and vomiting is still pyridoxine hydrochloride and doxylamine succinate, which has been shown to be effective in multiple randomized controlled trials. However, because several studies had small sample sizes, the therapeutic importance of these findings is still up for question. This suggests that larger trials are necessary to generate more conclusive evidence. Concerns about the safety of first-generation antihistamines and other pharmaceutical therapies, such as metoclopramide and ondansetron, have also been assessed; nonetheless, a major obstacle to their widespread usage is the possibility of teratogenic effects. These worries highlight how crucial it is for patients and professionals to jointly make decisions, carefully balancing the advantages and disadvantages of each available course of therapy. Furthermore, the use of intravenous fluids has been shown to effectively manage dehydration, and outpatient treatment options for intravenous fluid administration could potentially reduce hospital stays and costs, aligning with recent findings that emphasize the effectiveness of outpatient care for mild to moderate HG cases. These cost-saving approaches are important given the large economic burden of HG. For example, one national study estimated that HG costs healthcare systems approximately 5.2 million USD per year [93]. This underscores the financial as well as clinical importance of effective management.

Interestingly, some dietary interventions, particularly high-protein, low-carbohydrate diets, have shown promise in alleviating nausea and vomiting, but further clinical validation is crucial before they can be recommended as standard practice. The importance of *Helicobacter pylori* eradication in the treatment of HG is still unknown, despite the lack of evidence supporting its potential value in lessening the severity of symptoms. Similarly, additional research is required to fully understand the therapeutic potential of certain antidepressants and proton pump inhibitors in the context of HG, even if they may help manage particular symptoms like constipation and psychological distress.

Comorbid conditions like diabetes, epilepsy, and autoimmune illnesses can make managing HG more difficult, particularly if oral intake is impaired. Pregnant women who have previously undergone bariatric surgery may be even more susceptible to intestinal blockage and nutritional deficiencies, making the management of HG even more difficult. Special attention should be given to patients who have underlying medical conditions, and our results support the need for a comprehensive, tailored approach to therapy.

Lastly, it has been extensively shown that HG can reoccur in consecutive pregnancies, with recurrence rates ranging from 15% to 86%. Different study approaches may be the cause of this variability; studies conducted in hospitals typically indicate lower recurrence rates than cohort studies that rely on self-reported symptoms. Further research is necessary to determine the possibility of preventive measures such as early pyridoxine and doxylamine use, as some studies indicate that early management may lessen the intensity and length of symptoms. To validate the efficacy of this strategy and investigate additional potential preventive strategies, further thorough research is necessary. This aligns with recent evidence showing that early pharmacological intervention for NVP significantly improves maternal well-being and can reduce hospital admission rates [103]. These findings underscore the clinical importance of initiating treatment at the onset of nausea and vomiting, which may help prevent progression to severe HG.

Despite tremendous advancements in our knowledge of the etiology and treatment of HG, many questions still need to be answered. More investigation is required to better understand the mechanisms behind HG, improve treatment regimens, and create more potent therapies. Improving patient outcomes will require the combination of multidisciplinary techniques, such as dietary changes, psychological assistance, and pharmaceutical treatments. Developing customized care regimens for women with this crippling illness also requires investigating the effects of comorbidities and recurrence in later pregnancies.

As a narrative review, our synthesis is inherently subject to certain limitations. First, we did not conduct a formal risk-of-bias or quality assessment of included studies. Second, our literature search was restricted to English-language publications. This language limitation may have introduced selection bias and excluded valuable studies published in other languages. While this was necessary to ensure consistency and prevent translation-related errors, it nonetheless restricts the generalizability of our findings. Future systematic reviews would benefit from the inclusion of multilingual databases and cross-cultural perspectives to enhance global applicability.

Recent research has identified novel targets for HG therapy. For example, the GDF15–GFRAL signaling pathway is a key driver of HG symptoms, and experimental GDF15 antagonists show promise for alleviating nausea and weight loss. Other future approaches include targeting gut–brain peptides (e.g., ghrelin agonists) and using biomarker profiling to tailor treatment. These emerging molecular therapies and personalized medicine strategies warrant further investigation to improve HG management.

## 5. Conclusions

In conclusion, doxylamine–pyridoxine remains the first-line FDA-approved therapy for HG, while antihistamines (e.g., diphenhydramine), metoclopramide, or ondansetron serve as second-line options, and corticosteroids are reserved for refractory cases. Our review underscores the serious maternal risks of HG (including electrolyte disturbances, Wernicke’s encephalopathy, and thromboembolism) and adverse perinatal outcomes (preterm birth and fetal growth restriction). We emphasize early recognition and a multidisciplinary approach—combining pharmacotherapy, nutritional support, and psychosocial care—to optimize outcomes. Finally, ongoing research into molecular targets (e.g., GDF15 antagonists) and individualized therapies offers promise for future management. These conclusions directly reflect the evidence and discussions presented above.

## Data Availability

No new data were created or analyzed in this study.

## Abbreviations

The following abbreviations are used in this manuscript:

HG	Hyperemesis Gravidarum
NVP	Nausea and Vomiting of Pregnancy
FDA	Food and Drug Administration
hCG	Human Chorionic Gonadotropin
GDF15	Growth Differentiation Factor 15
TNF-α	Tumor Necrosis Factor-Alpha
H. pylori	Helicobacter pylori
WE	Wernicke’s Encephalopathy
CPM	Central Pontine Myelinolysis
DVA	Developmental Venous Anomaly
RCT	Randomized Controlled Trial
CS	Cohort Study
CaS	Case Study
OS	Observational Study
DS	Descriptive Study
SCS	Single-Center Study
RT	Randomized Trial
PICOS	Population, Intervention, Comparison, Outcome, Study Design
CSS	Cohort Study (retrospective)
HIV	Human Immunodeficiency Virus
